# Radiographic study of the distribution of maxillary intraosseous vascular canal in Koreans

**DOI:** 10.1186/s40902-015-0045-x

**Published:** 2016-01-04

**Authors:** JuHyon Lee, Nara Kang, Young-Mi Moon, Eun-Kyoung Pang

**Affiliations:** 1 Department of Oral and Maxillofacial Surgery, College of Dentistry Jukjeon Dental Hospital, Dankook University, Yongin, South Korea; 2 Department of Oral and Maxillofacial Surgery, Kunkuk University Medical Center, Seoul, South Korea; 3 Department of Conservative Dentistry, College of Dentistry Daejeon Dental Hospital, Wonkwang University, Deajeon, South Korea; 4 Department of Periodontology, Graduate School of Medicine, Ewha Womans University, 1071, Anyangcheon-ro, Yangcheon-gu, Seoul, 158-710 South Korea

**Keywords:** Maxillary artery, Posterior superior alveolar artery, Sinus augmentation

## Abstract

**Background:**

This study aimed to investigate the distribution and prevalence of intraosseous loop (anastomosis between posterior superior alveolar artery and infraorbital artery) in Koreans detected on computed tomography (CT) images taken prior to sinus augmentation surgery.

**Methods:**

From the 177 patients who underwent sinus augmentation with lateral approach at Ewha Womans University Department of Implant Dentistry, 284 CT scans were evaluated. The canal height (CH), ridge height (RH), and canal height from the sinus floor (CHS) were measured on para-axial views at the first premolar, first molar, and second molar. The horizontal positions of the bony canals in the lateral wall were also classified. One-way analysis of variance (ANOVA) and *t* test were used to estimate the statistical differences (*p* < 0.05).

**Results:**

The intraosseous loops were detected in 92 CT scans (32 %). The mean vertical height of the bony canals from the alveolar crest (CH) was 23.45 ± 2.81, 15.92 ± 2.65, and 16.61 ± 2.92 mm at the second premolar, first molar, and second molar, respectively. In the horizontal positions of the bony canals, intraosseous type was the most predominant. The canal heights more than 15 mm and less than 17 mm were most prevalent (33.7 %) and those under 13 mm were 12.0 %.

**Conclusions:**

The radiographic findings in this study could be used to decide the lateral osteotomy line avoiding potential vascular complication. However, only one third of the canals could be detected in CT scans; a precaution should be taken for the possibility of severe bleeding during lateral osteotomy.

## Background

Sinus augmentation with lateral osteotomy is a predictable surgical technique that allows successful placement of dental implants to patients with extremely atrophic posterior maxilla. Despite the high level of safety and predictability [1–3], severe vascular complications may occur during lateral osteotomy as a result of arterial injury [4, 5]. Therefore, knowledge of the arterial supply of the maxillary sinus region is crucial to avoid untoward complications [6, 7].

The arterial supply of the maxilla originates from the posterior superior alveolar artery (PSAA) and infraorbital artery (IOA). PSAA is the first branch of the third portion of the maxillary artery (MA) and usually arises just before the MA enters the pterygopalatine fossa [8]. PSAA divides into intraosseous branch (IObr) and extraosseous branch (EObr) before entering the posterior superior alveolar foramen. Each branch forms an anastomosis with IOA and creates intraosseous loop and extraosseous loop [7, 9].

The IOA frequently arises from a common trunk with the PSAA and runs anteriorly along the roof of the maxillary sinus (orbital floor). Within the orbit, it gives rise to muscular branches as well as the anterior superior alveolar arteries, which forms an anastomosis with the PSAA [7–11].

The intraosseous loop, an anastomosis between the IObr of the PSAA and IOA, was found in 100 % of anatomic specimens at the lateral antral wall, 18.9 ± 2.82 mm from the crestal margin [7, 9]. Elian et al. examined 50 computed tomography (CT) scans of the maxillary sinus from 625 patients and detected the bony canal (intraosseous loop) in 53 % of cases [12].

Owing to its location, the intraosseous loop has the potential to cause bleeding during lateral window osteotomies [7, 9, 11, 12]. Thus, it is clinically important to detect the intrabony course of the vessels for planning the proper osteotomy line to avoid damage to the vessels and to maintain perfusion of the entire bone segment [9]. However, few studies of the distribution of the intraosseous loop have been done, with none involving Koreans. The purpose of this study was to investigate the distribution and the prevalence of the intraosseous loop in Koreans detected on CT images taken prior to sinus augmentation surgery.

## Methods

This study was approved by the Ewha Womans University ** Hospital IRB (EUMC. 2014-08-009), and all participants signed an informed consent agreement.

### Materials

CT images from 177 patients (284 scans) who underwent sinus augmentation with a lateral approach at the Department of Implant Dentistry, Ewha Womans University from January 2002 to December 2008 were evaluated. In case of bilateral sinus augmentations, each sinus was counted separately. In CT images, only maxillary para-axial images were included (patients who did not have para-axial reconstruction were excluded). The axial cuts at 1-mm intervals were reconstructed into 3-mm cross sections (para-axial). Of 177 patients and 284 sinus CT scans, 68 (109 scans) were women and 109 (175 scans) were men; age ranged from 33 to 78 years (mean 55.9 years). Sixty (21 %) of the cases were fully edentulous and 224 (79 %) were partially edentulous. The right and left sinuses comprised 138 (51.4 %) and 146 (48.6 %) cases, respectively.

### Methods

**Fig. 1:**
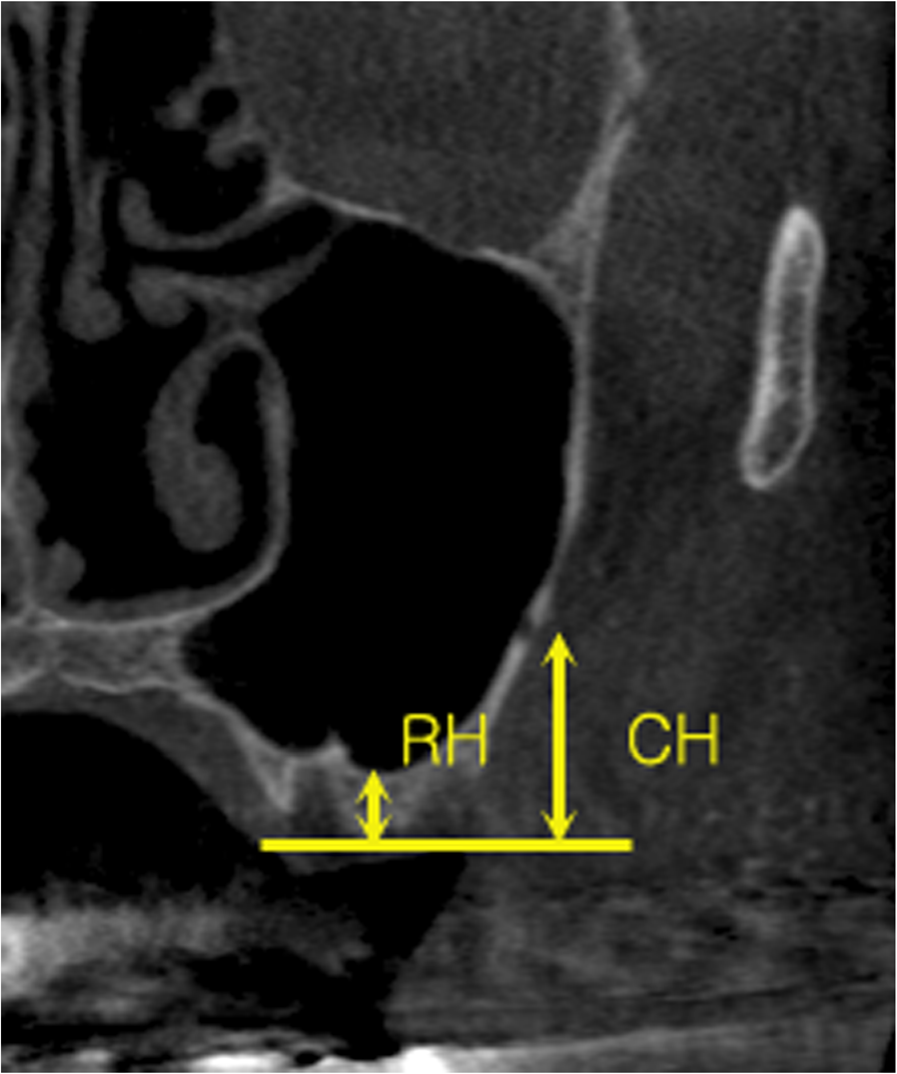
The vertical position of the bony canals according to the tooth region on para-axial sinus CT scan. *CH* (canal height), distance between alveolar crest and inferior border of the canal; *RH* (ridge height), distance between alveolar crest and sinus floor; *CHS* (canal height from the sinus); *CH*-*RH*

**Fig. 2:**
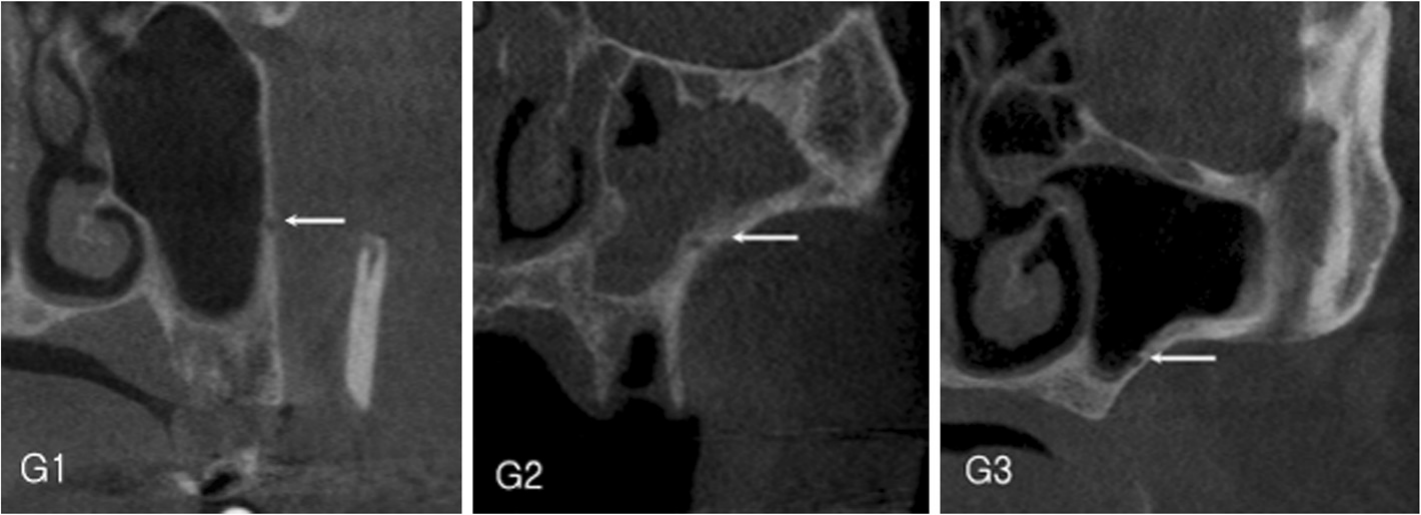
The horizontal position of the bony canals. *G1* extrasinusal, *G2* intraosseous, *G3* intrasinusal

Para-axial CT images were evaluated for the presence of a bony canal of the intraosseous loop in the lateral sinus wall. The canal height (CH) and ridge height (RH) were measured on para-axial cut using a digital caliper. CH defined a vertical distance from the alveolar crest to the inferior border of the canal. CH from the sinus (CHS) was calculated by subtraction of RH from CH. CH was measured at each tooth position from the first premolar to the second molar (Fig. 1). The horizontal position of the bony canals in the lateral wall mesiodistally from the sinus was classified into three categories: G1, in which the canal bulged towards outside of the wall (extrasinusal); G2, with the canal embedded in the sinus wall (intraossoeous); and G3, in which the canal bulged towards inside of the wall (intrasinusal) (Fig. 2).

### Statistical analysis

The measured values according to the tooth region and age were analyzed by one-way analysis of variance (ANOVA), and those according to the gender, edentulous/partial edentulous, and right/left were analyzed with *t* test (*p* < 0.05).

## Results

**Fig. 3:**
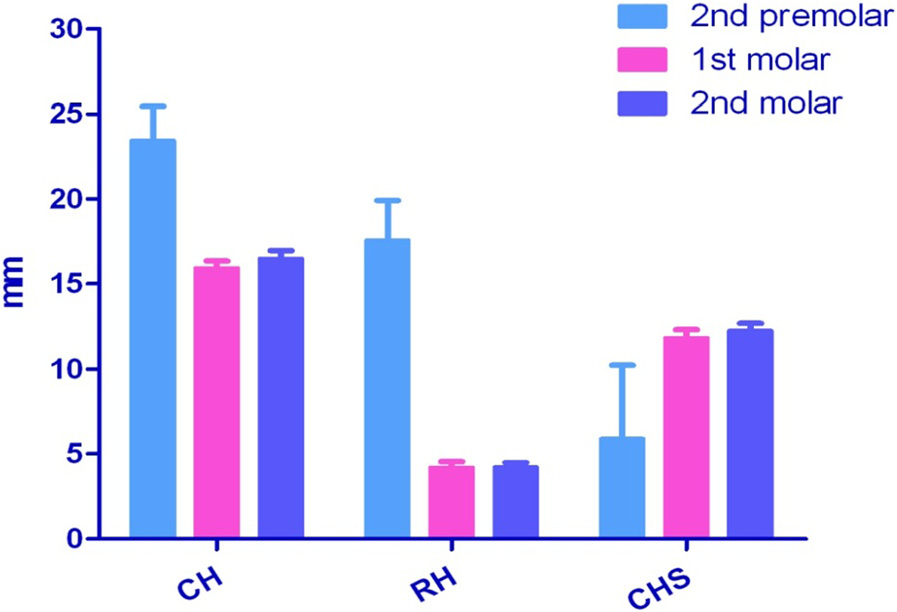
The vertical position of canals according to the tooth region

Two hundred eighty-four CT images were examined. The bony canal of the intraosseous loop was identified in 92 images (32 %). Concerning the vertical canal position according to the tooth region, the canal was observed to run most inferiorly from the alveolar crest at the first molar. CH from the sinus floor increased as the canal passed the posterior region (Table 1, Fig. 3). No significant differences were evident among the measured values according to the tooth region. The G2 (intraosseous) type of horizontal canal position was the most predominant in the lateral wall in the first molar and second molar regions (Table 2). The measured CH and RH values and calculated CHS values according to age, gender, edentulism, and right/left side are summarized in Tables 3 and 4. There were no statistical significances between the groups. CHs exceeding 15 mm and less than 17 mm were most prevalent (33.7 %), followed by CH over 19 mm (18.5 %) and CH under 13 mm (12.0 %) (Table 5).

**Table 1:** The vertical position of the bony canals according to the tooth region (mean ± SD, mm)

Tooth region	2nd premolar	1st molar	2nd molar
Identified/investigated	2/152 (1.3 %)	44/264 (16.7 %)	86/247 (34.8 %)
CH	23.45 (±2.81)	15.92 (±2.65)	16.61 (±2.92)
RH	17.56 (±3.30)	4.19 (±2.15)	4.21 (±1.86)
CHS	5.89 (±6.11)	11.83 (±3.16)	12.21 (±2.87)

*CH* canal height (distance between alveolar crest and inferior border of the canal), *RH* ridge height (distance between alveolar crest and sinus floor), *CHS* canal height from sinus (CH-RH), *G1* extrasinusal, *G2* intraosseous, *G3* intrasinusal

**Table 2:** The horizontal position of the bony canals according to the tooth region

Tooth region	Number	G1	G2	G3
2nd premolar	2	2 (100 %)	–	–
1st molar	44	8 (18.18 %)	34 (77.27 %)	2 (4.55 %)
2nd molar	86	–	75 (87.21 %)	11 (12.79 %)

**Table 3:** Canal height according to age (mean ± SD, mm)

Age	30s	40s	50s	60s	70s
Identified/investigated	6/18	17/61	36/86	25/95	8/24
CH	18.06 (±4.11)	15.48 (±3.01)	16.49 (±2.87)	16.45 (±3.51)	16.75 (±2.82)

**Table 4:** Canal height according to gender, edentulism, and right/left (mean ± SD, mm)

	Gender		Edentulism		Right/left	
	Female	Male	Full	Partial	Right	Left
Identified/investigated	31/109	61/175	18/60	74/224	45/138	47/146
CH	16.88 (±3.67)	16.18 (±2.85)	16.76 (±3.41)	16.36 (±3.10)	16.85 (±3.04)	16.25 (±3.49)

**Table 5:** Distribution of canal height (mean ± SD, mm)

CH	<11	<13	<15	<17	<19	<21	21≤	Sum
*N* (number)	2	9	17	31	18	9	6	92
*N* (%)	2.2	9.8	18.5	33.7	19.6	9.8	6.4	100

## Discussion

A bony canal of the intraosseous loop at the lateral wall was presently identified in 32 % of the examined CT images. The mean canal height was 23.45 ± 2.81, 15.92 ± 2.65, and 16.61 ± 2.92 mm at the second premolar, first molar, and second molar, respectively. The determined mean heights between the canal and alveolar crest of the particular tooth area was shorter than 50 % of those previously reported in radiographic studies [12, 13]. The reconstruction of CT images in the 3-mm interval para-axial cut cannot be ruled out as the reason for the shorter prevalence because the CT images were reconstructed from 1-mm interval para-axial cuts in other studies.

In a cadaveric study, the intraosseous loop was always found at the lateral wall of the anatomic specimens while the extraosseous loop was found only in 44.4 % near the periosteum at the level of 22.75 ± 1.49 mm from the alveolar crest [7, 9]. In a Korean anatomic study, the intraosseous loop was also found in all examined cadavers; the average external diameter was 0.9 ± 0.3 mm and the mean height from the CEJ was 24.1 ± 4.6, 21.1 ± 4.8, and 22.4 ± 3.7 mm in the second premolar, first molar, and second molar, respectively [10]. These results were much higher than those of the present study. The previous study had a reference point on the CEJ of the tooth, while the present study set a reference point on the alveolar crest of edentulous region. Alveolar bone resorption after extraction could be an explanation of the presently higher results. In the present study, as well as in anatomic studies [7, 9, 10, 14] and a radiographic study [13], the intraosseous loop formed a concave arch, with the first molar area being the lowest point of the bony canal arch course. During surgical procedures including lateral window osteotomy, more precautions should be taken at the first molar region than at the premolar region.

The most frequent horizontal position of the canal in the lateral wall was intraosseous or intrawall type (G2), 77.27 % in the first molar and 87.21 % in the second molar region. The results corresponded with another radiographic study [15], but in Korean cadavers, the canals were observed in G1 position most frequently [10].

Hur et al. [10] and Elian et al. [12] recommended designing the superior osteotomy line 13 to 15 mm from the alveolar crest for placing proper length of dental implant [10, 12]. In this study, a CH exceeding 13 mm was evident in 88 % of CT scans. This result indicates that 12 % of the cases could be followed by surgical vascular complications.

Even though the intraosseous loop was radiographically evident in only 32 % in this study, the results could be used to prevent the arterial bleeding at the time of lateral window surgery, especially under local anesthesia.

## Conclusions

In the horizontal positions of the bony canals, the intraosseous type was most predominant. CHs more than 15 mm and less than 17 mm were most prevalent (33.7 %) and those under 13 mm were 12.0 %. The radiographic findings in this study could be used to decide the lateral osteotomy line avoiding potential vascular complication. However, since only one third of the canals could be detected in CT scans, precautions should be taken for the possibility of severe bleeding during osteotomy.
